# Transgenerational Transmission of Trauma across Three Generations of Alevi Kurds

**DOI:** 10.3390/ijerph19010081

**Published:** 2021-12-22

**Authors:** Jan Ilhan Kizilhan, Michael Noll-Hussong, Thomas Wenzel

**Affiliations:** 1 Institute for Psychotherapy and Psychotraumatology, University of Duhok, Zakho Street, 1006AJ Duhok, Iraq; 2 Psychosomatic Medicine and Psychotherapy, Saarland University Medical Centre, Kirrberger Straße 100, D-66421 Homburg, Germany; michael.noll-hussong@uks.eu; 3 World Psychiatric Association Scientific Section on Psychological Aspects of Persecution and Torture, CEHRI and OEGBA, 1090 Vienna, Austria; drthomaswenzel@web.de

**Keywords:** psychological trauma, transgenerational transmission, genocide

## Abstract

Background: Thus far, most researchers on genocide and transgenerational transmissions have focused on the National Socialist Holocaust as the most abhorrent example of this severe human rights violation. Few data have been published on other ethnic or religious groups affected by genocidal actions in this context. Methodology: Using a mixed-method approach integrating qualitative interviews with standardized instruments (SCID and PDS), this study examines how individual and collective trauma have been handed down across three generations in an Alevi Kurd community whose members (have) suffered genocidal perpetrations over a longer time period (a “genocidal environment”). Qualitative, open-ended interviews with members of three generations answering questions yielded information on (a) how their lives are shaped by the genocidal experiences from the previous generation and related victim experiences, (b) how the genocidal events were communicated in family narratives, and (c) coping strategies used. The first generation is the generation which directly suffered the genocidal actions. The second generation consists of children of those parents who survived the genocidal actions. Together with their family (children, partner, relatives), this generation suffered forced displacement. Members of the third generation were born in the diaspora where they also grew up. Results: Participants reported traumatic memories, presented in examples in this publication. The most severe traumatic memories included the Dersim massacre in 1937–1938 in Turkey, with 70,000–80,000 victims killed, and the enforced resettlement in western Turkey. A content analysis revealed that the transgenerational transmission of trauma continued across three generations. SCID and PDS data indicated high rates of distress in all generations. Conclusions: Genocidal environments such as that of the Kurdish Alevis lead to transgenerational transmission mediated by complex factors.

## 1. Introduction

Unfortunately, extreme violence and human rights violations remain a common or even increasing challenge in many countries and are followed by a massive impact on group and individual mental health [1]. In general, trauma can be understood as the effect of an overwhelming and extraordinary experience that leaves its victim feeling helpless [2]. This feeling of powerlessness can manifest itself in several or all realms of life and functioning of a person who has been traumatized, namely, general self-efficacy, the ability to cope with stressful events, and the ability to build or maintain close personal relationships. The vulnerability of traumatized persons to the development of specific and nonspecific trauma-related symptoms such as those of depression, anxiety, somatoform disorders, affective reaction disorders, psychosis, substance abuse, and post-traumatic stress disorder (PTSD) is considered high [3,4], and therefore these are also summarized as trauma spectrum disorders [5]. Symptoms observed after exposure to extreme violence such as concentration camp experiences have also earlier been described as the concentration camp survivor syndrome by Bower and others [6], encompassing a number of complex symptoms not completely covered, though overlapping, with present diagnostic categories. In addition to the severe consequences for those who were directly affected, others who were not directly exposed to this event, especially family members, may also suffer from complex consequences [7,8], sometimes termed second-generation syndrome [9] if applied to the offspring of first generation survivors.

This can also apply to whole groups, as defined by ethnicity and religious affiliation, who suffer collective trauma as the whole group is directly threatened and persecuted [10]. Both the individual and the collective are victimized and experience a rupture in their daily routine that is passed on from one generation to another and is constantly being transformed [11].

A growing body of publications has explored possible direct and indirect mechanisms of transgenerational transmission, and, most recently, epigenetic mechanisms [12]. Through research on the Jewish Holocaust, the impact of parental trauma on offspring has also been well demonstrated [9,13,14]. The term “Intergenerational transmission” could be used in this context [15], as causal mechanisms are still the subject of discussion, as we will show below. In the following part of our introduction, we want to shortly summarize the most relevant concepts without aiming at giving a comprehensive review. 

Coping and adaptation patterns are passed on to future generations and can be integrated as part of culture, religions, and norms and can be experienced through communication in the form of oral tradition, but also through rituals and ceremonies [16]. This process of passing on group behavior patterns, but also parent–child transmission [17], including the interruption of healthy, “normal” parenting and transmission patterns [9], is carried out on both a conscious and unconscious level [18,19]. Kellermann (2001), based on social-learning theory, distinguished transference, affection, punishment, and overprotection as parenting behaviors of second-generation survivors. Resilience in some groups and individuals, as well as the respective role of direct and indirect mechanisms, are further subject of discussion. 

In this context, most theories focus on the psychological transference mechanisms of family patterns at both conscious and unconscious levels [14]. Williamson and Bray (1988) proposed that family patterns are developed and transmitted through the process of projection both within the nuclear family emotional system and across generations [14].Other perspectives suggest the development and transmission of emotional themes which are shaped through loyalties and ties between family members [20]. Such loyalties are considered a fundamental and essential source of trust. It has been suggested that they determine the individual’s behavior through their subconscious characteristics. They manifest themselves in a considerable commitment to the family of origin and to the individual’s parents if they are present [21]. 

In order for the older generation to be able to come to terms with the trauma experiences, the children consciously and subconsciously try to take over emotional themes from them in an attempt to relieve them. However, the emotional themes can be assumed to be the children’s own emotions if they are not sufficiently reflected and sustained. These processes are based on transgenerational patterns and imperatives which, in turn, rely on two fundamental conditions. First, the children perceive an emotional debt to parents. Additionally, there is the differentiation of the self. Both conditions are considered an indication that the children have been unable to resolve emotional attachments to the parents [22].

Williams and Bray (1988) assume, on the basis of their studies, that children take over certain emotional themes of the older generation, so that this generation can find a conclusion through children, or at least a compensation takes place. Certain issues within a family are in turn reproduced by the children because the older generation in particular cannot deal with the stressful experiences.

According to this view, the subjectively experienced emotional guilt towards the survivors plays a role, which is why the following generations take over the passing on of these experiences themselves and are not able to deal with them sufficiently [22]. Thus, this leads to over-identification with parents or the older generation and their experienced burdens to invisible loyalties, and emotional attachment with a never-ending settlement of multigenerational debts. This can lead to a persistent and strong emotional attachment to the family in some children, resulting in a sacrifice of autonomy and selfhood [22]. 

In connection to investigations in this realm, there are several problems. First, there is a general lack of actual research supporting the respective models. Additionally, the scarcity of studies coincides with several methodological problems. These difficulties include the validity and reliability of test instruments as well as samples and a failure to address gender-specific aspects [23]. However, the few existing research results can be considered a significant reference. According to these results, it at least can be seen as certain that traumatic experiences from war contexts are handed down to the subsequent generations [24,25]. 

Studies have found the following common themes for second- and third-generation transference phenomena: increased awareness of parents’/grandparents’ status as (Jewish) Holocaust survivors, parenting style, over-identification with parents’/grandparents’ experiences, and general fear and distrust of people. 

Other studies emphasize internal psychological conflicts as a consequence of a protective role which children of Holocaust survivors take on. These conflicts emerge from an incompatibility between feelings of responsibility to those who were killed during the Holocaust and a feeling of having failed because one was not able to save the victims. These studies conclude that the conflicts lead to mental illness [26].

In contrast, the studies of Hantman (Hantman & Solomon, 2007) and Juni (2016) assume that the third generation is better able to cope with the experiences of their grandparents compared to the second generation. They indicate higher levels of psychological well-being by the third generations. The authors assume that, compared to the second generation, grandchildren have a better self-understanding and are better able to cope with their grandparents’ experiences from a certain distance. Thus, the second generation is assumed to have a higher level of effect than the second generation [27,28].

Studies on other ethnic and religious groups that have experienced genocides and massacres have come to different conclusions. These investigations include studies on the emergence of transgenerational trauma from the 1915 Armenian Genocide [29,30]. These examinations observed a growing number of psychological disorders across two generations. It was found that the third generation suffered psychological disorders more frequently than the previous generation. Similar findings can be seen in the study by Rosenheck and Fontanas (1998) which focused on Vietnam veterans. Their fathers were World War II veterans. It could be shown that the third generation suffered trauma and displayed a higher number of psychological disorders [31]. 

Based on current studies, it can be in conclusion assumed that, according to the concept of the transgenerational transmission of traumatization, parental traumatization at least potentially affects offspring who were not yet born at the time of the traumatic event [11,32]. According to some authors, vulnerability in respect of the emergence of mental disorders is passed on [33,34] through mechanisms such as projection and identification [35] as well as at the psychophysiological or epigenetic [36] level [17]. 

In this context, historical traumas that also date back several generations seem to be important for the psychological burden of their descendants. Consequently, we want to take a closer look at the topic of transgenerational trauma, in particular despite, or precisely because of, the still-developing and preliminary conceptualization of the subject and the lack of research on other genocides besides the better-explored Jewish Holocaust (Kellermann, 2013).

### Historical Trauma of the Population of Dersim and the Situation of Kurdish Alevis as Background to the Study

As a concrete case study for many groups, the historical and current traumas of the Kurdish Alevis from Turkey can lead to a better understanding of transgenerational trauma. 

The Alevis make up approximately ten to a maximum of twenty percent of the population of Turkey. Of these, about one-third are Kurdish-speaking Alevis who live in eastern Turkey [37]. Alevis differ from both Sunni and Shiite Islam in their religious practices and beliefs. In the Alevi faith of eastern Anatolia, there are some motifs of Shiite mythology but also some ancient Mithraic rituals and ceremonies that do not exist in Islam [38].

Alevi ritual and religious practice is strongly influenced by oral tradition. There is no central Alevi authority and no unified written corpus. Authority in Alevism is embodied in the figure of the *“dede”* (Turkish for grandfather). A “dede” is the male representative of a lineage that are revered as sacred and who alone can lead the Alevi ritual and who, in traditional Alevism, were responsible for the religious and social leadership of Alevi communities [39] 

In 1937–1938, a military campaign took place against parts of Tunceli province, formerly Dersim, Turkey. The military operation was officially “justified” with the argument that the Kurdish population and rebels were “out of control” [40]. The operation lasted from March 1937 to September 1938 and, according to official figures, claimed 13,160 lives. A total of 11,818 persons were resettled (Radikal 20 November 2009). The people themselves and contemporary witnesses speak of between 30,000 and 70,000 deaths and the forced resettlement of some 20,000–50,000 people. In a speech in November 2009, the current President and then-Prime Minister Recep Tayyip Erdoğan spoke of a “massacre” and referred to more than 13,000 deaths [40,41,42]. The Dersim campaign was prepared well in advance and was not a short-term reaction to a specific uprising. According to the Turkish government in the 1940s, it was intended to control the ever-growing Kurdish population through assimilation, forced resettlement, and military operations [43]. The Dersim area is mainly inhabited by Kurdish Alevis.

## 2. Aims of This Study

In our study, we wanted to explore possible links between historical factors (such as major events still present in the memories of group members) with present factors (such as recent events serving as triggers for memory recall) in the defined ethnic group of Kurdish Alevis, who were exposed to generations of persecution and genocidal action in a continuous (genocidal) environment. We further intended to better understand the complex interaction of coping, stressors, and communication patterns in the families in regard to traumatic past events and intergenerational transmission of memories.

## 3. Methodology and Methods

Due to the limitations of the present conceptualization of the field, and the lack of clearly defined syndromes or diagnostic categories, and corresponding diagnostic standardized instruments, we decided to use a mixed-method approach, integrating qualitative research to explore possible reaction patterns, symptoms, and mechanisms in addition to well-established standard instruments as described in more detail below. 

Inclusion criteria: We included representatives of three successive generations of Kurdish Alevi families fulfilling the following criteria: (1) the first generation had been personally threatened, had several family members killed, had seen people killed themselves and had had to flee or had been forcibly resettled; (2) all families had to be Kurdish Alevis and originally from Dersim; (3) family members of all the families that are survivors of the Dersim massacre of 1937–1938, in which officially more than 13,000 community members were killed [43]. 

Sample recruitment: Sample recruitment needed to be conducted with care due to the ongoing persecution and was based on purposive sampling, using a network of trusted key informants including relatives, acquaintances, and friends who were treated by the author in a transcultural psychosomatic clinic in Germany and Alevi associations in Germany. Security concerns were considered in this process.

The interviews were conducted in Germany by the first author (JIK) and the interviews in Turkey by two psychologists. Psychologists had received comprehensive training to conduct quantitative and qualitative interviews and documentation.

### 3.1. Sample Description

Based on these criteria, we included the following group of participants: 

Eight participants, aged between 9 and 13 during the traumatic event in 1937–1938, but now elderly, experienced the massacre, and were forcibly relocated with their families to western Turkey. Ten participants and their families belong to the second generation, who were born and grew up in western Turkey. They still live in western Turkey. Twelve other participants belong to the third generation and were born and raised in Germany.

*Procedures:*

*Qualitative component:*

An interview guideline was developed, discussed with the interviewers and corrected, and the final version accepted. The interviews for the qualitative part of the survey were recorded, transcribed, and rendered anonymous by four trained psychologists.

Each participant was contacted and asked by the author to give informed consent. Written consent was obtained from all participants after the study had been explained.

The participants were visited in their home by the trained psychologist and completed the interview face-to-face. Interviews were recorded on a digital audio device, pseudonymized, transcribed, and analyzed on a PC. Evaluation was conducted according to qualitative content analysis after Mayring [44]. The analysis and building of categories were supported by Atlas.ti 5.2.12 (ScientificSoftware Development GmbH). Categories were developed inductively from the material in an interplay with the theory (research questions). The interviews were analyzed on the basis of a code system with the following steps: selection of units for analysis, definitions of the dimensions for structuring, definition of the characteristics and development of the code system, description of definitions, examples and rules for coding, coding, extraction of the codes, revision of the code system and further coding and interpretation, and preparation of results. In order to meet the quality criteria of qualitative research, in particular intersubjective comprehensibility [29], two master’s degree students at the university developed main categories and sub-categories from the responses inductively and independently of each other. The classifications were compared and discussed from the point of view of a consensual coding. This procedure ensured both inter-subjectivity and comprehensibility of the results [44]. Major categories and subcategories were discussed with the two students during consensus meetings until mutual agreement was reached. The author (JIK) re-coded the material according to the final categories.

The ethics board of the University of Duhok in Northern Iraq approved the study. 

The following test instruments were used for the quantitative part of the study to assess different relevant social and clinical aspects.

### 3.2. Sociodemographic Characteristics

The sociodemographic data and context characteristics in connection with the work and the personal trauma or flight experiences were assessed by a questionnaire developed for the study.

*Quantitative component (clinical aspects):*

*Structured clinical interview (SCID)*

The Structured Clinical Interview for the Diagnostic and Statistical Manual of Mental Disorders (DSM; SCID) is a widely used semi-structured interview intended to determine whether an individual meets the criteria for any DSM disorder [45]. We used the version adapted for DSM-IV. The Turkish version of the instrument has been previously used with Turkish-speaking populations and was found to be appropriate for this population [46].

The interview was performed to determine the consequences war trauma on the mental health of survivors (APA, 2015). This allowed, in this study, diagnosis of any mental disorders of three generations after the genocide in 1937–1938 against Alevi Kurds according to the DSM-IV classification relevant at that time [45].

### 3.3. Posttraumatic Diagnostic Scale (PDS)

The Posttraumatic Diagnostic Scale (PDS) [47] was used as a quantitative method to determine the severity of PTSD symptoms, and has been used in a number of studies on trauma-affected groups [48]. PDS is a self-assessment questionnaire that measures the frequency of the 17 PTSD symptoms mentioned in DSM-IV on a four-step scale. Mean values of the characteristic can be calculated for the three symptom complexes ‘intrusions’, ‘avoidance’, and ‘hyperarousal’. The re-test reliability in the original version is r = 0.74 for the total score, r = 0.66 for the cluster of intrusive symptoms, r = 0.56 for the avoidance symptoms, and r = 0.71 for the hyperarousal.

### 3.4. Statistical Analysis

The authors used means, percentages, and distributions for the sample description. Mann–Whitney-U-tests for independent samples were used for the analysis of the differences in means, since the data were not normally distributed. To test the correlations of determinants with the respective outcome (PDS scores), the Spearman rho test was applied as a nonparametric measure. Multiple-linear-regression analyses were performed to assess whether the determinants were associated with the PDS scores and to identify potential risk and resilience factors. The level of significance for all analyses was set at α = 0.05. All statistical analyses were performed using IBM SPSS Statistics version 24. As no validated statistical norm data are available in the ethnic group in question, we restricted analysis mainly to descriptive methods.

## 4. Results

### 4.1. Sample Composition

In the first generation (n = 8), the mean age was 89 years, with a balanced percentage of 50% men and 50% women. All participants had at least five years’ schooling and had been employed in low-level jobs.

In the case of the second generation (n = 10) living in west Turkey, the sample consisted of 60% men and 40% women, with a mean age of 63 years. Four participants had a university degree, three participants had a secondary-school degree, and three had a college diploma. In the third generation (n = 12) who were all born and living in Germany, the sample consisted again of 50% men and 50% women, with a mean age of 41 years. Of the participants, four had a university degree, another five had a high-school diploma, and all were employed in a professional capacity. Three participants had a graduate degree and were employed as workers.

The study did not show any significant differences between responses given to the male and female interviewers (psychologists). This applies to all results of this study, which consistently yielded no gender differences. 

### 4.2. Diagnostic Evaluation

In the diagnostic examination with the SCID by trained clinical psychologists, a larger part of the first-generation sample was diagnosed with affective disorders (65%), followed by anxiety disorders (52.5%) and somatoform disorders (36.3%). In the second generation, affective disorders (38.5%) were again the most common diagnosis, followed by anxiety (26%) and somatoform disorders 834.5%). In the third generation, personality disorders were diagnosed in 12%, in addition to affective disorders (18.5%), anxiety (16%), and somatoform disorders (11.5%). Symptoms of PTSD could be observed in participants of the second and third generation, as summarized in Table 1, although they were not directly affected by the genocide or severe trauma.

Thus, all participants of the first generation (100%) reported a personal trauma history, 25% of the second, and 16.7% of the third generation reported a trauma history. At least 50% of the first generation, 25% of the second generation, and 16.7% of the third generation reported a traumatic experience. All participants of the first generation report experience of forced migration (100%), while 16.7% of the second generation report a forced migration. The third generation, who were born in Germany, did not have a personal history of flight, but had grown up in a more stable environment. 

The analysis of the interviews conducted with three generations allowed the development of a system of categories. These categories cover several questions. First, they address the way how those affected remembered the massacres perpetrated in 1937 and 1938. Moreover, they record how the massacre events were communicated. In connection to that, the frequency of mention was documented. Finally, the strategies which enabled the first generation and the subsequent generations to cope with the massacre events and to develop resilience were addressed. This category system, in turn, could be used to show mechanisms of how trauma was handed down to the subsequent generations and mechanisms of how those affected could cope with transgenerational trauma by drawing on resilience, for example.

As part of the “genocidal environment” experienced, present events and ongoing narratives triggered memories and aggravated the feeling of continuous danger (Table 2).

First-generation representatives commented most frequently on repressive measures they had experienced (all participants, i.e., 100%), the flight from their place of origin (all), and religious persecution (87.5%). The struggle of the Kurds in Turkey (75%), the politics of the government in Turkey (62.5%), international events such as wars and flight (62.5%), and the death of relatives (62.5%) still occupy their minds.

Table 2 shows that the second generation mainly commented most frequently on the struggle of the Kurds in Turkey (80%), government policy (80%), and events (80%) leading to the memory of the massacre in Dersim. 

Statements by the participants described the immediate experience of this continuous persecution (genocidal environment):

*The government continues to deny the Dersim massacre and we Alevis continue to be discriminated against…. We are Kurds and Alevis, that is the reason. It is not getting better.*

(Participant 1)

The third generation in particular described religious persecution (58.3%), the Kurdish struggle in Turkey (58.3%), political organizations (41.7%), and events (41.7%). For more than a third of the third generation, the death of relatives (36.7%) was also an issue of concern.

*The Alevis are and still are called infidels by the Sunnis. My family had to hide the fact that they are Alevis for a long time. During fasting days, we got up in the night even though we do not fast like the Sunnis. We turned on the lights at night so that the neighbors would think that we were also starting the fast.*

(Participant 12)

### 4.3. Types of Communication

The sub-categorization scheme below shows the relevance of the modes of communication used between the survivors and the following generations. The ways of communication encompass a wide range of communication. On the one hand, there was regular communication, which means that those affected openly addressed potentially traumatic events in everyday conversations. On the other hand, unspoken assumptions, silence, taboo, and secrets were identified as ways of communication as well. From these insights, it can be deduced that the mode of communication significantly shapes the way members of the subsequent generations integrate trauma experienced by their parents or grandparents with their lives. Table 3. gives a summary of the communication patterns observed in members of the different generations.

The communication style of the first generation was less open (25%) compared to the second generation (50%), while the second (60%) and third (58.3%) generations tended to communicate about the massacre through music and written and oral narratives.

The first generation largely preferred the mode of indirect communication (75%). An overwhelming majority considered avoidance, tabooing, or silence as most appropriate ways of approaching the topic of the massacres from 1937 and 1938 (87.5%). However, this attitude decreased in the subsequent generations. While it persisted among half of the second-generation participants, it could be observed to a significantly smaller extent (16%) among the third generation.

*I do not talk about the massacre with my children, or only a few if they ask questions. I do not want to stress them and I want to protect them.*

(Participant 1)

An interviewee reported that their parents had never told them directly about their personal experiences during the massacre in 1937–1938. The traumas of their parents or grandparents were unconsciously communicated, for example, when they listened to their parents’ or grandparents’ conversations with other elderly Alevis and realized that they must have experienced similar or related catastrophic events.

*My parents did not talk about Dersim. I always learned what really happened from friends, at events, and later through books. They never really answered my question. They always said it was bad and you do not need to know about it… For me, it was terrible that I did not know what had happened to them and that they were always so sad.*

(Participant 18)

Guilt, shame, and the fear that their experience would negatively affect the life of the subsequent generations frequently characterized the communication patterns of the first generation. This often led to silence, secrecy, and unspoken issues related to traumatic experiences and the historical event of the massacre. Since the grandparents did not talk about their traumatic experiences, it was difficult, especially for the second, but also for the third generation, to integrate these events psychologically and biographically, although such a process is significant for identity. 

Nevertheless, the experiences and processing of trauma were valued very differently by the three groups: 50% of the first generation, 60% of the second, and 33% of the third generation try to avoid the topic. Identity and trauma seemed to be strongly linked, which was significant for the sense of lack of belonging to a country or place among the third generation (58%).

*Why should we talk about something that is already over. The dead do not come back to life; the pain does not never pass. There is no need to talk about it. We just have to forget about it.*

(Participant 8)

The constant remembrance of trauma and its visualization was most frequent among the first generation (87.5%). Experiences of guilt, victimization, and subjugation were reported by 62.5% of the first generation, 70% of the second and, somewhat surprisingly, by 33.3% of the third generation.

*I want to forget, but I cannot. I remember every day how they slaughtered us and the river turned red from the blood.*

(Participant 1)

The second and third generations reported feelings of anxiety and symptoms of restlessness and tension in the PDS questionnaire, as well as in the interviews, reflecting a sense of imminent threat, as if they had experienced their parents’ traumatic events themselves or had lived during the time when these events had occurred.

*The Dersim massacre is not over. After that, the government and radical Sunnis continued. They attacked and killed hundreds of Alevis in their homes in the city of Maras in 1978… I will never forget the scenes on TV when radical Sunnis, with the help of the government, set fire to a hotel where Alevi artists and academics were holding a conference in 1993. We must always be on guard that nothing happens to us. I do not trust myself to say everywhere that I am an Alevi*.

(Participant 12)

The qualitative analysis of the interviews reveals five main ways of processing the massacre perpetrations from 1937/1938 and of coping with coinciding traumatic experiences. These strategies were considered effective in all three generations. First, those affected attempted to attach meaning to the events by ascribing the survivors’ story to a mystical sense of uniqueness. Additionally, those affected felt able to process and cope with the experience by visiting the places where massacres were perpetrated. Moreover, music and art were considered effective coping strategies. In addition to that, social cohesion and unity were perceived as essential to encounter the challenges caused by the massacre experiences. This attitude was relatively consistent across the different generations (75%/60%/75%). Finally, similar developments could be observed for the conviction that their religion is a universal as well as peaceful faith and therefore an effective coping mechanism (62.5%/50%/60%).

*I have been playing saz … (musical instrument) … since I was a child and I have memorized all the songs about the massacre. It is good for me to deal with the pain, even if it is sad. Through music and my religion, I get support to be able to live on.*

(Participant 14)

The strong connection between the second as well as third generation with the surviving (great) grandparents is reflected in similar ways of communication and of approaching and coping with issues. The trauma events of their parents and grandparents have an important impact on the development of their lives and continue to influence their cognitions, emotions, and behavior.

## 5. Discussion

*Limitations:* The selection of participants and the small sample size can be seen as an obstacle to generalization, though they are a common factor in qualitative research. Results can be seen as a pilot for similar studies in this and other groups exposed to war and genocidal environments. 

### Benefits of the Study and Discussion of Results

Despite its small sample size and its partial exploration of trauma and strategies which allow those affected to cope with massacre events, this study is valuable contribution in the several ways. First, the use of a mixed-method approach and of qualitative interviews have enabled a better understanding of how traumatic experiences and coping strategies are handed down across different Kurdish Alevite generations and integrates the aspects of established diagnostic categories with the more open and flexible qualitative interview, which thus far has been rarely used in the field of genocide studies which usually has been restricted to either one of the two approaches. 

Fundamentally, the study has shown that traumatic experiences arising from the 1937/1938 massacres have been handed down across three generations. In connection to that, it can be pointed out that the negative experiences which were directly suffered by the first generation have had a continuing and a significant, negative impact on the subsequent second and third generations, as also observed for example by other authors using qualitative models [25]. The negative consequences manifest themselves in the similar key topics which are connected to the traumatic events in each family and which have been reflected in the interviews with each generation. From this, it can be deduced that traumatic events which are severe and experienced by ancestors can significantly and extensively affect the subsequent generations. Data from the standardized PTSD symptom questionnaire (PDS) further indicated a slow reduction in symptoms over generations, but persistence in some participants, which would indicate the importance of identification and special support of these especially vulnerable persons.

Moreover, this study suggests that how a trauma is handed down across generations depends on the question of what exact kind of trauma was experienced. Additionally, this study has revealed that the familial behavioral patterns and the particular life mission of a family in one generation were handed down from that generation to the subsequent generations. In other words: the second and third generation did not question the attitude from previous generations, adopted ideas, and considered them self-evident and fixed.

In the families, loyalty as a form of survival strategy and of not forgetting seemed to have been passed on to the descendants [10] as a form of inheritance. The families talk openly about this.

Although the massacre is not openly discussed in the survivors’ families and sometimes the survivor generation is silent, they do communicate through music, stories, and various repetitive events and rituals. It seems clear that the wounds of the Dersim massacre will never heal, but must be recalled and memories kept alive—and this out of loyalty. It seems that this kind of tradition is caused by a feeling of guilt which is focused on the experiences suffered by the first generation. The tradition functions as a familial lesson. It must be taught to the subsequent generations. In this way, the tradition can be seen as a kind of familial ‘mission’. 

The way the survivors process the traumatic events psychologically is connected to the tradition with which they hand down their experiences to the subsequent generations (communication style). The findings in this study reflect the results from previous studies (Braga, Mello, & Fiks, 2012) focusing on samples from other (ethnic) groups insofar as these previous studies emphasized the impact of emotional control, narrative, and symbolic representation on how subsequent generations approach the trauma experienced by their (grand)parents (Barga et al., 2012). It must be pointed out that traumatic experiences are deeply entrenched in the second and third generation, particularly when members of the previous generation hand down these experiences in an indirect way by choosing to taboo the experiences and remain silent on them. The way in which the traumatic experiences are passed on by survivors can significantly affect how subsequent generations process these experiences psychologically.

Among other things, this form of communication can lead to the second and third generations (who did not experience the massacre) linking their own emotional and psychosocial deficits to the inability of their grandparents to adequately overcome the trauma. In this way, the experiences suffered in 1937 and 1938 can be perpetuated, which affects the psychological well-being of the subsequent generations extensively. Additionally, the experiences can be perpetuated in various other ways. Those affected can suffer from feelings of guilt which can be caused by external signs or symbols. Symbols can also prompt feelings of becoming the object of victimization or subjugation. In all these scenarios, the worldview of those affected is characterized by fear and distrust. The behavioral and emotional patterns which can be observed among those connected to traumatic stress are a logical and therefore normal consequence if the knowledge about the traumatizing experiences is communicated by a significant other [7]. Previous studies (Figley 1995; Kizilhan & Noll-Hussong 2017) have pointed out that the reaction to trauma which had been handed down by previous generations becomes evident in secondary traumatic stress disorder. This disorder, in turn, manifests itself in symptoms which parallel those of PTSD. This reflects the findings of previous studies on this topic [49], which found that PTSD clearly became evident in the second and third generations of the survivors’ families. In connection, the emergence of PTSD did not depend on the question of how immediately or directly the traumatic experiences were suffered. Members of subsequent generations stated they suffered from nightmares. These nightmares were characterized by scenarios in which those affected were chased by soldiers. Moreover, scenes of massacre perpetrations, flight, and expulsion constituted the majority of the nightmares. While describing these nightmares, those affected showed strong emotional reactions. 

On the basis of the cases studied here, the current political situation of the Kurds and Alevis, and their discrimination, seem to lead to a perpetuation and transmission of the trauma. As a result, in addition to the degree of secondary traumatization, the current life situation (political, social, religious, and economic), and denial of the state, also influence the psychological state of all three generations [39,50].

Moreover, the extent to which traumatic experiences are handed down can also be determined by the individual and the degree to which the individual is emotionally vulnerable and resilient. Previous work has pointed out that different individuals can show different, individual reactions to traumatic events (i.e., the severity of parental trauma). Additionally, previous studies have shown that extensive trauma can overwhelm predispositions [51]. Apart from personal conditions, social resources must be considered as well when looking at the questions of how reactions to traumatic experiences are shaped [52]. These perspectives find expression in the model by Danieli who suggests that one can consider the vulnerability and resilience of an individual in a framework with several dimensions which manifest themselves in multiple levels of post-traumatic adjustment [11]. These considerations suggest that the extent to which traumatic experiences are passed on would vary between individuals and families. In the light of these insights, the limitations of the results in this study become evident, as the sample size is very small and because the percentage of participants from each generation is relatively low. 

However, it is possible that the consequences of the traumatic experiences result in the emergence of resilient patterns among subsequent generations. This can happen when members of a particular generation defend universal or community values, when they are socially or politically active, and when they look for collective attachment and social support networks. Members link the traumatic experiences to these activities and, in this way, they develop resilience [53].

In their attempts to process more knowledge about the massacre and the trauma, the third generation favors, among other things, artistic creation and implementation processes (music, poetry, novels, narratives, etc.). This can be understood as a legacy, in that the following generations have taken up this event as a part of their collective memory and, through different forms of communication, do not wish to forget it. 

For the third generation, living in migration, this transgenerational trauma is also a coping strategy, enabling them to develop a positive perspective integrating with their identity of origin and migration as a transcultural identity [7,10,31].

As with any qualitative or mixed method study, the findings here cannot be generalized to all members of the group or other groups, as noted in the list of limitations given earlier. This type of research is usually small scale and exploratory in nature. It would be interesting to expand its scope to other settings and to compare not only the descendants of war victims, but also other minorities such as the Yazidi, Kakai, Ismaili, Bahai, Uighur and Kazakhs, or Oriental Christians in the Middle East, who have also experienced numerous historical traumas. The gender-specific aspects of trauma processing and its transmission are also topics for further research. The description of some specific aspects found in the results can also be explored in further research using complementary methods such as quantitative approaches.

## 6. Conclusions

The findings suggest that older generations affect the psychological well-being of subsequent generations in different ways by passing on traumatic experiences to subsequent generations. The impact on subsequent generations can manifest itself in PTSD symptoms. However, the former is not limited to the latter. This highlights the need for physicians, therapists, social workers, and other professional groups in contact with survivors to be aware of the trauma-related background of their clients and its possible impact, even though the client may not present it as relevant. It is necessary for clinicians to understand clients’ perceptions of their grandparents’ and parents’ trauma-related past, the general genocidal environment of their group, and their role in relationships within the family. In this sense, the findings from this study on the transmission of trauma in families and other collectives may be worth further investigation in order to re-evaluate transgenerational, collective, and individual trauma in terms of their appropriateness and impact on subsequent generations’ own lives and to consider these when treating their clients. 

When considering family narratives, which are connected to a particular trauma, one must pursue and approach the following questions. First, one must identify the mechanisms with which subjectively perceived obligations (i.e., family mission) are handed down to subsequent generations. Moreover, one must pose the question of what the situation looks like after the family mission was accomplished. Up to now, there are no insights into whether the members and families of a particular generation find another mission or whether the mission is characterized by a different direction. Moreover, it is worth asking whether the family mission can be considered a commitment to become the object of commemoration, when the mission requires a concrete action and that action is completed. Future studies must address these and other questions. Doing so, future work must focus on how these questions are related to effects and characteristics of how the trauma is handed down across generations.

## Figures and Tables

**Table 1:** PTSD symptom levels (measured by PDS) and experiences of trauma among the three generations.

PDS	Re-Experiencing the Event n (%)	Avoidance n (%)	Hyperarousal n (%)	Repercussions of the Preceding Symptoms on Activities of Daily Life n (%)	Personal Experience of Traumatic Events (Flight, War, Violation, etc.) n (%)
Whole Sample	12 (40.0)	13 (43.3)	11 (36.7)	9 (30.0)	13 (43.3)
First Generation	6 (75.0)	4 (50.0)	5 (62.5)	4 (50.0)	8 (100.0)
Second Generation	4 (33.3)	5 (41.7)	4 (44.3)	3 (25.0)	3 (25.0)
Third Generation	2 (16.7)	4 (33.3)	2 (16.7)	2 (16.7)	2 (16.7)

PDS = Posttraumatic Diagnostic Scale.

**Table 2:** Events which reportedly evoke memories of the massacre of 1937–1938 in our group.

	1st Generation (n = 8)		2nd Generation (n = 10)		3rd Generation (n = 12)		M	χ^2^ df = 2
	n	%	n	%	n	%	%	
**International events**	5	62.5	5	50.0	4	33.3	46.7	1.7311 *
Kurdish struggle in Turkey	6	75.0	8	80.0	7	58.3	70.0	1.3458 **
Religious persecution	7	87.5	7	70.0	7	58.3	71.9	2.1056 **
Flight from country of origin	8	100.0	3	80.0	0	0.0	60.0	n. b.
Experienced repression	8	100.0	5	50.00	4	33.3	56.7	n. b.
Political organizations	3	37.5	6	60.0	5	41.7	46.7	1.1095 *
Government policy	5	62.5	8	80.0	4	33.3	56.7	5.1845 **
Death of relatives	5	62.5	4	40.0	2	16.7	36.7	4.5708 *
Events and gatherings (e.g., anniversaries, festivities, funerals, etc.)	5	62.5	8	80.0	5	41.7	51.1	3.4870 *

M = mean frequency; n. b. = not calculable; * *p* < 0.05; ** *p* < 0.01.

**Table 3:** Ways of communicating following the study of Braga et al., 2012.

Communication	1st Generation n = 8	2nd Generation n = 10	3rd Generation n = 12	M	χ^2^ df = 2
	n (%)	n (%)	n (%)	%	
Type of Communication					
Open, everyday communication	2 (25.0%)	5 (50.0%)	4 (33.3%)	36.7%	1.2928 *
Communication via formal records and documents (music, remembrance events, written and oral story-telling)	3 (37.5%)	6 (60.0%)	7 (58.3%)	53.3%	1.1095*
***Indirect communication***					
Catastrophic, fragmented communication	6 (75.0%)	4 (40.0%)	3 (25.0%)	46.7%	5.1003 *
Secrets, silence, and taboos	7 (87.5%)	7 (50.0%)	2 (16.7%)	46.7%	10.7507 **
**Experience of trauma**					
Attempts to suppress memories of the catastrophe (avoidance)	4 (50.0%)	6 (60.0%)	4 (33.3%)	47.8%	1.6285 *
Identity: lack of roots and a feeling of belonging	2 (25.0%)	5 (50.0%)	7 (58.3%)	47.8%	2.2945 **
Envisioning the traumatic experience	7(87.5%)	5 (50.0%)	4 (33.3%)	53.3%	6.2878 *
Experiencing guilt, victimization, and subjugation	5 (62.5%)	7 (70.0%)	4 (33.3%)	55.3%	3.3768 **
The struggle to have the injustice recognized	3 (37.5%)	5 (50.0%)	7 (58.3%)	50.0%	0.8402 *
Attempts to explain their survival and the effects on subsequent generations	5 (62.5%)	4 (40.0%)	5 (41.7%)	46.7%	1.1095 *
**Mechanisms of psychological processing and resilience**					
Search for a mystic sense of uniqueness of the survivors’ (hi)story	6 (75.0%)	5 (50.0%)	5 (41.7%)	53.3%	2.2945 **
Seeking out places which are linked to the traumatic experiences of the survivors	6 (75.0%)	4 (40.0%)	4 (33.3%)	46.7%	3.7215 *
Art and culture as a possible means of representing the disaster	2 (25.0%)	7 (70.0%)	9 (75.0%)	51.1%	5.6700 **
Loyalty, bonding, and social support of the peer group	6 (75.0%)	6 (60.0%)	9 (75.0%)	70.0%	0.6892 *
Defending the Alevi religion as a universal, humanist, and peaceful faith	5 (62.25%)	5 (50.0%)	8 (66.7%)	60.0%	0.6564 **

M = mean frequency; n. b. = not calculable; * *p* < 0.05; ** *p* < 0.01.

## Data Availability

Data supporting reported results can be found in the safe server of the main author’s University.
